# Identification of Small Molecule and Genetic Modulators of AON-Induced *Dystrophin* Exon Skipping by High-Throughput Screening

**DOI:** 10.1371/journal.pone.0008348

**Published:** 2009-12-17

**Authors:** Debra A. O'Leary, Orzala Sharif, Paul Anderson, Buu Tu, Genevieve Welch, Yingyao Zhou, Jeremy S. Caldwell, Ingo H. Engels, Achim Brinker

**Affiliations:** Genomics Institute of the Novartis Research Foundation, San Diego, California, United States of America; National University of Ireland Galway, Ireland

## Abstract

One therapeutic approach to Duchenne Muscular Dystrophy (DMD) recently entering clinical trials aims to convert DMD phenotypes to that of a milder disease variant, Becker Muscular Dystrophy (BMD), by employing antisense oligonucleotides (AONs) targeting splice sites, to induce exon skipping and restore partial dystrophin function. In order to search for small molecule and genetic modulators of AON-dependent and independent exon skipping, we screened ∼10,000 known small molecule drugs, >17,000 cDNA clones, and >2,000 kinase- targeted siRNAs against a 5.6 kb luciferase minigene construct, encompassing exon 71 to exon 73 of human *dystrophin*. As a result, we identified several enhancers of exon skipping, acting on both the reporter construct as well as endogenous *dystrophin* in mdx cells. Multiple mechanisms of action were identified, including histone deacetylase inhibition, tubulin modulation and pre-mRNA processing. Among others, the nucleolar protein NOL8 and staufen RNA binding protein homolog 2 (Stau2) were found to induce endogenous exon skipping in mdx cells in an AON-dependent fashion. An unexpected but recurrent theme observed in our screening efforts was the apparent link between the inhibition of cell cycle progression and the induction of exon skipping.

## Introduction

Duchenne Muscular Dystrophy (DMD) is the most common of nine categories of muscular dystrophy, occurring at an incidence of 1/3500 live born males [1]. All cases of DMD are caused by a loss of dystrophin protein expression, however the underlying genetic mutations for the disease vary greatly between individuals and can include deletions, insertions or point mutations throughout the *dystrophin* gene (*DMD*), which is the largest gene in the human genome (spanning 2.4 Mb of the X chromosome) [2], [3]. The severity of muscle wasting in DMD means that most patients die in the second decade of their lives due to respiratory and cardiac failure, as a consequence of loss of dystrophin expression in both cardiac and skeletal muscle [4].

Existing DMD therapies are limited to symptomatic treatments such as glucocorticoids, which decrease inflammation resulting from muscle cell necrosis and degeneration [5], and improve muscle strength in DMD patients and tissue engineered from mdx mice (carrying a spontaneous point mutation in *Dystrophin*) [6], via as yet unknown mechanisms. While lifespan and quality of life can be slightly improved through these treatments [7], the underlying genetic defect remains. Small molecules that may prove beneficial to DMD patients include histone deacetylase (HDAC) inhibitors. Treatment with Trichostatin A (TSA) can improve morphology and function of skeletal muscle in mdx mice via the upregulation of follistatin [8], and valproic acid can improve muscle integrity and function in the mdx/Utrophin^−/−^ double mutant mouse model of DMD via activation of the Akt pathway [9], however these compounds are yet to be tested in humans. A small molecule showing potential for treating a subset of DMD patients with nonsense mutations is PTC124. Efficacy studies in humans are currently ongoing, following successful studies in the mdx mouse [10], and safety and tolerability in a phase I trial [11].

One therapeutic approach currently pursued in the clinic that could treat up to 83% of all DMD cases [12] attempts to convert DMD to BMD phenotypes. BMD is a milder and rarer form of muscular dystrophy (∼1/20,000) [13] caused by mutations in *dystrophin* that enable the production of partially functional truncated protein products [14], [15]. AONs can be designed against splice sites or enhancer elements to induce exon skipping in cells of DMD patients, and have shown restoration of the reading frame of dystrophin 28 days after intramuscular injection of AON into the tibialis anterior muscle [16]. Further clinical trials are underway to test different AON chemistries and specific sequences targeting exon 51, as this AON alone could treat 13% of DMD patients [12], [17]. Studies have shown that as little as 29% of normal levels of dystrophin protein can alleviate symptoms of muscle weakness [18], however there has been limited success of restoration of dystrophin expression in the heart following intravenous administration of AONs in the mdx mouse [19], [20], unless given every other day, over several days or weeks [21], [22]. Regular intramuscular or intravenous injection is cumbersome and is yet to be tested in DMD patients for its impact on muscle tissue integrity. An additional disadvantage to AON-based therapy of DMD is the need to personalize AON sequences depending upon the patient's specific *dystrophin* mutation.

Given the limitations of existing and experimental treatments, there remains an unmet clinical need for the development of small molecule therapeutics for DMD. Moreover, there is evidence for the existence of endogenous mechanisms enabling exon skipping within *DMD* transcripts that contain nonsense [23]–[25] or frameshift mutations [26]. This highlights an opportunity to identify novel therapeutic targets for the treatment of DMD and other genetic diseases. In this study we aimed to identify small molecule and genetic enhancers of AON-dependent and independent exon skipping through the screening of small molecule libraries with annotated functions, in addition to cDNA and siRNA collections. Besides several expected mechanisms of action, and a number of new genetic modifiers including NOL8 and Stau2, these screens revealed an unexpected connection between the inhibition of cell cycle progression and enhancement of *DMD* exon skipping. This general trend hints at a potentially novel mechanism of action for HDAC inhibitors in DMD treatment.

## Results

### DMD Minigene Construct Features Spontaneous Exon Skipping

**Figure 1:**
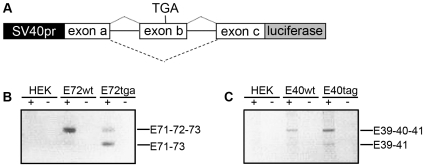
Splice forms and spontaneous exon skipping of *DMD* minigene reporter constructs in HEK 293 cells. (A) Schematic of *DMD* minigene *luciferase* reporter gene constructs. A genomic segment of human or mouse *DMD* containing three exons (a, b, and c) and their intervening introns (solid lines) was cloned downstream of the SV40 promoter and in-frame with the *luciferase* coding sequence of the pGL3 vector. Dotted lines represent splicing of full-length transcript and dashed lines that of the exon skip transcript. Variations of each construct were made containing stop codon mutations in exon b, as indicated by TGA. (B) RT-PCR products with primers spanning *DMD* exon 71 to *luciferase* (marked + or − for with and without reverse transcriptase enzyme) from untransfected HEK 293 cells or cells transfected with the hE72-Luc construct with (tga) or without (wt) a TGA stop codon mutation, showing spontaneous exon skipping that is enhanced upon addition of a nonsense mutation. (C) RT-PCR products with primers spanning *DMD* exon E39 to *luciferase* from untransfected HEK 293 cells or cells transfected with the hE40-Luc construct with (tag) or without (wt) a TAG stop codon mutation show the same effect, although less dramatic.

The large size of the *DMD* gene (79 exons spanning 2.4 Mb), limits the ease of generating genomic overexpression constructs. However, since splicing can involve enhancer and repressor sequences within introns [27]–[29], and pre-mRNA secondary structures within introns can influence exon recognition [30], [31], we decided to generate luciferase reporter gene constructs spanning three exons with full-length intervening intronic sequences to enable protein-protein interactions of all necessary splicing factors (Figure 1A). Two genomic fragments of human *DMD* were selected for generation of luciferase reporter gene constructs based upon three characteristics: 1) their ability to be cloned by conventional means (<20 kb in size), 2) the generation of an in-frame transcript as a result of exon skipping and 3) a report of patients carrying stop codon mutations within the central exons (to allow for the generation of minigene constructs with low basal activity and which mimic mutations documented in the Leiden DMD mutation database) [32].

Two resulting minigene constructs spanned the 5′ end of exon 71 to the 3′ end of exon 73 (hE72-Luc) and the 5′ end of exon 39 to the 3′ end of exon 41 (hE40-Luc). Although these constructs did not encompass the mutation hotspot regions between exons 2–20 or 45–53, truncation mutations 5′ of exon 74 are known to invariably cause DMD [33], [34]. This observation is probably a result of nonsense-mediated decay of transcripts or a lack of functional actin or β-dystroglycan domains in the resulting protein [32]. Reports have been made of spontaneous exon skipping for some DMD and BMD patients with stop codon mutations [24], [25], [35], including within exon 72 [23]. When we transiently transfected HEK 293 cells with either hE72-Luc(TGA) or hE40-Luc(TAG) construct this phenomenon could be reproduced *in vitro* and detected by RT-PCR (Figure 1B–C). Sequencing and quantification of relative densities of RT-PCR products confirmed significant enhancement of skipping of exon 72 in the presence of a stop codon mutation (45.1% of transcripts from the hE72-Luc(TGA) construct versus 16.5% of transcripts generated from hE72-Luc(WT)). Similar results were seen with hE72-Luc(TAG) and hE72-Luc(TAA) constructs (data not shown). In contrast, introduction of a stop codon mutation only minimally increased exon skipping of the hE40-Luc construct (26% of transcripts from hE40-Luc(TAG) versus 24.6% of transcripts from hE40-Luc(WT), indicating that sequence specific splicing factors may be involved. Together these results validated the two *DMD* minigene constructs, in that they could mimic the phenomena of endogenous exon skipping reported in DMD patients with stop codon mutations, and that they could be used to identify small molecule and genetic regulators of these endogenous processes without having to artificially induce the process with high levels of AON.

Transient transfection of minigene constructs in HEK 293 cells was used for genomic and small molecule compound high-throughput screens (HTS) described below, due to superior luciferase signal when compared to stable clones in either HEK 293 or C2C12 cellular backgrounds (data not shown). Use of HEK 293 cells was not expected to limit the hits obtained from our screens, given that spontaneous exon skipping of minigene constructs could occur in this cell line, and that an analogous screen monitoring splicing of a *microtubule-associated protein tau (MAPT)* construct was recently performed in HEK 293 cells and identified drugs capable of functioning on endogenous *MAPT* in SHSY-5Y neuroblastoma cells [36]. Reporter gene assay conditions were further optimized by testing the concentration of AON and identifying a positive control reference compound.

### Validation of DMD Reporter Gene Assay

**Figure 2:**
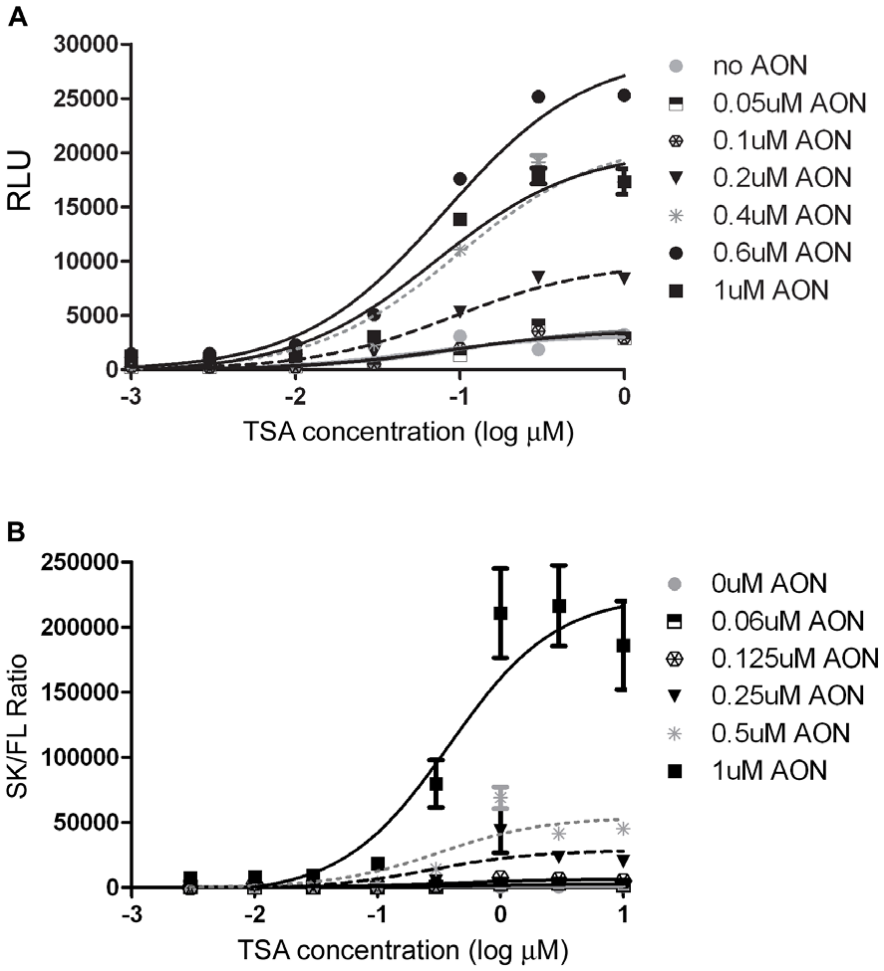
TSA enhances AON-induced exon skipping of hE72-Luc *DMD* minigene reporter construct in HEK 293 cells. (A) HEK 293 cells were transiently transfected with hE72-Luc and 0–1 µM hE72 AON 24 h prior to addition of 0–1 µM TSA in 1536-well format. Luciferase activity (RLU = relative luciferase units) was measured 24 h after compound addition (48 h post-transfection) and shows a positive trend with AON concentration that is enhanced with TSA treatment. Inhibition of signal begins to occur at 1 µM AON (black squares). Data points are an average of 6 replicates and error bars represent standard deviation. (B) HEK 293 cells were transiently transfected with hE72-Luc and 0–1 µM hE72 AON 6 h prior to addition of 0–10 µM TSA in 24-well format prior to qPCR analysis of transcripts 24 h after compound treatment. An increase in the ratio of exon skip (SK) to full-length (FL) transcripts in seen with increasing AON concentration that is enhanced with TSA treatment, indicating it is not a general increase in transcriptional activity. Data points are an average of 5 replicates and error bars represent standard deviation.

hE72-Luc(TGA) was chosen for HTS as it offered a 10 times higher baseline luciferase signal than hE40-Luc constructs (presumed to be due to higher rate of spontaneous exon skipping of hE72-Luc(TGA)), allowing for a more robust assay, while still giving relatively low luciferase expression prior to AON or compound treatment (RLU ∼20% of saturation point of detection system). Additional reporter gene constructs hE40-Luc(WT) and mE23-Luc(TAA) (mimicking the mdx mutation in exon 23 of mouse *Dmd*) were used to probe for sequence-specificity of reconfirmed screen hits. Given the evidence that HDAC inhibitors can enhance both general transcription and specific splicing of genes such as *survival of motor neuron 2 (SMN2)* [37] and *cystic fibrosis transmembrane conductance regulator (CFTR)* [38], we tested a panel of compounds of this class against hE72-Luc(TGA) (data not shown). TSA was found to give the greatest signal increase and was therefore chosen as a positive control for the small molecule screen. Since we wished to identify both AON-dependent and independent genes and small molecule compounds, we titrated a validated 2′O-Methyl AON specific to *DMD* exon 72 (hE72 AON) [39]. By analyzing a titration matrix of AON and TSA (Figure 2A) a limiting dose of 0.5 µM hE72 AON was chosen for all further experiments, to allow for basal luciferase expression and detection of small molecule and genetic enhancers of AON-induced exon skipping, in addition to AON-independent regulators. The hE72-Luc reporter gene assay was further validated by qPCR. Primer-probe sets were designed to amplify transcripts containing human exon 71-exon 73 (exon skip) and exon 72-exon 73 (full-length) *DMD* splice junctions. Data from each primer-probe set was normalized to expression levels of the h36B4 housekeeping gene for each sample and results then expressed as the fold change in the ratio of normalized skip/full-length transcript levels (Figure 2B). At each dose of hE72 AON tested, TSA was seen to give an additional increase in exon skip transcript levels, indicating that it was not simply enhancing transcriptional activity in general in the context of the hE72-Luc reporter. Thus the qPCR assay highlights two separate mechanisms for TSA to enhance the luciferase signal of the DMD minigene construct – one as a general effecter of gene transcription by modulating chromatin structure, and another as a modulator of pre-mRNA splicing. Given its large assay window and mechanistic relevance, 1 µM TSA was used as a positive control during the small molecule screen.

### Tubulin Modulators and HDAC Inhibitors Enriched in Known Drug Hit List

A collection of ∼10,000 “known drug” small molecule compounds with functional annotation derived from public databases including PubChem and the World Drug Index (WDI) [40], was screened at a single dose of 8.3 µM (0.83% DMSO) in 1536-well format against HEK 293 cells transiently transfected with the hE72-Luc(TGA) construct and 0.5 µM hE72 AON. This screen yielded a Z' = 0.6, indicating a robust assay amenable to HTS. The concentration of compound used was the highest dose possible in 1536-well format, since DMSO concentrations above 1% resulted in cellular toxicity (data not shown). From this screen 70 compounds gave ≥2 fold increase in luciferase signal above plate mean values in duplicate (0.70% hit rate) and 66 of these showed a dose-response. From this hit list 37 unique compounds (several structures were represented more than once due to duplication in the library screened) showing sigmoidal dose-response curves were chosen for re-testing from purified powders (>85% purity by LC-MS) in 384-well format, allowing for dosing up to 100 µM. 21/37 compounds reconfirmed from powder, in addition to the positive control TSA. The two most common classes of compound identified from this small molecule screen were tubulin modulators and HDAC inhibitors (Table 1), the most potent being apopicropodophyllin-beta and podophyllotoxin, cyclolignans known to inhibit microtubule assembly [41]. Although the majority of reconfirmed compounds significantly increased the mitotic index of HEK 293 cells and showed toxicity at equivalent or lower concentrations than the EC50 in hE72-Luc reporter gene assay, qPCR analysis demonstrated that 17/21 of the compounds specifically enhanced exon skipping in the context of the hE72-Luc reporter construct. Those compounds giving a skip/full-length transcript ratio of >2 after normalization to DMSO treated cells were regarded as specific enhancers of exon skipping.

**Table 1:** Small molecule compounds with reconfirmed activity in hE72-Luc assay^[a]^, their known functions, cell cycle stage of action, activity and potency in hE72-Luc, HCI mitotic index and Alamar blue toxicity assays in HEK cells, as well as exon skipping activity on the hE72-Luc construct in HEK cells and on endogenous mouse *Dystrophin* transcripts in mdx cells, as determined by qPCR.

Common name	Known Function	Cell Cycle Stage of Action	hE72Luc+AON Median FC^[b]^	hE72Luc+AON Potency (EC50 in µM)	hE72+AON qPCR^[c]^	mdx+AON qPCR^[d]^	mdx qPCR^[e]^	Mitotic Index Median FC	Mitotic Index Potency (EC50 in µM)	Toxicity (IC50 in µM)
Apopicropodophyllin-beta	Tubulin modulator	G2/M [41]	3	0.03	22.86	<2	3.56	14	0.02	0.04
Colchicine	Tubulin modulator	G2/M [92]	2	1.5	27.83	<2	3.66	8	0.05	0.2
Demecolcine	Tubulin modulator	G2/M [92]	3	1	9.59	<2	<2	11	0.0004	0.001
Fenbendazole	Tubulin modulator	G2/M [93]	3	2.2	2.76	2.21	<2	6	0.1	0.4
Mebendazole	Tubulin modulator	G2/M [93]	4	4.5	3.87	2.17	<2	10	0.3	0.3
Nocodazole	Tubulin modulator	G2/M [94]	8	0.94	12.49	<2	<2	7	0.02	0.02
Oxibendazole	Tubulin modulator	G2/M [95]	3	3.2	3.45	<2	3.29	9	0.2	0.2
Podophyllotoxin	Tubulin modulator	G2/M [41]	5	0.16	21.09	<2	<2	11	0.0003	0.0003
Tn-16	Tubulin modulator	G2/M [96]	18	6.1	7.55	2.83	2.57	12	0.2	0.06
Tubulazole	Tubulin modulator	G2/M [97]	10	1.2	25.88	<2	6.93	8	0.05	0.07
Tubulin polymerization inhibitor	Tubulin modulator	G2/M [97]	3	0.96	4.13	3.09	6.92	5	4.7	10
Dinaline	HDAC Inhibitor	G1 [98]	56	10	3.20	2.18	3.68	<2	NA^[g]^	12
Nicotinamide analog	HDAC Inhibitor	ND^[f]^	4	65	9.98	<2	4.90	15	0.7	0.1
Scriptaid	HDAC Inhibitor	G1+G2/M [99]	47	0.54	16.79	<2	4.95	2	0.8	2.7
Trichostatin A	HDAC Inhibitor	G1+G2 [100]	17	0.16	45.51	<2	4.03	<2	NA^[g]^	0.3
Doxifluridine	DNA intercalator	S [101]	4	6.2	5.53	17.01	3.12	<2	NA^[g]^	0.03
Hoechst	DNA intercalator	S [102]	32	1.3	31.79	<2	2.43	<2	NA^[g]^	2.5
Luteolin	Flavonoid	G1 [103]	3	6.5	<2	3.11	4.21	<2	NA^[g]^	80
Pin1 modulator	Kinase inhibitor	G2/M [104]	3	3.3	3.97	2.30	2.77	11	0.3	0.5
Steroid receptor modulator	Receptor modulator	ND^[f]^	9	9.3	<2	2.43	2.49	6	3	8
Parthenolide	Anti-inflammatory	G2/M [105]	11	10	<2	2.08	2.87	3	1.5	10
Diphenyleneiodonium	Flavoenzyme inhibitor	G1+G2/M [106], [107]	3	8.5	<2	10.02	<2	<2	NA^[g]^	1

[a] Compound classed as reconfirmed hit when luciferase activity of purified powder was >2 fold above DMSO in duplicate plates; [b] FC = fold change above DMSO; [c] assay was performed in the presence of 0.5 µM AON and 1 µM compound in HEK cells transfected with hE72-Luc and data represents average fold change in the normalized skip/full-length ratio; [d] assay was performed in the absence of AON and presence of 1 µM compound in mdx-H2K cells and data represents average fold change in the normalized skip/full-length ratio; [e] assay was performed in the presence of 0.2 µM AON and presence of 1 µM compound in mdx-H2K cells and data represents average fold change in the normalized skip/full-length ratio; [f] not determined; [g] not applicable.

Next we wished to test whether the 21 reconfirmed hit compounds had the ability to modulate exon skipping of endogenous *Dmd* transcripts in mdx cells. While the expression of DMD protein significantly increases upon *in vitro* differentiation of healthy muscle cells into multinucleated myotubes [42], the variable nature of differentiation within and between experiments made study in myotubes infeasible for medium-throughput dose-response studies. Concurrent qPCR studies of DMSO-treated mdx myoblasts and myotubes showed that full-length *Dmd* transcript levels were not significantly different pre- and post-differentiation for 3 days (P = 0.2, student's two-tailed t-test), and more reproducible results were obtained from myoblasts (than myotubes) upon treatment with increasing concentrations of AON and TSA controls, so all further studies were conducted in mdx myoblasts. qPCR showed that 16 of the reconfirmed compounds also induced exon skipping of endogenous mouse *Dmd* exon 23 in mdx cells in the absence of mE23 AON (skip/full-length ratio >2 using primer-probes specific to exon 22–24 and exon 22–23 splice junctions). In mdx cells only 8/16 of these compounds induced exon skipping at higher levels than 0.2 µM mE23 AON alone. This is in part due to the high potency of the AON (0.2 µM mE23 AON induces 200–600% increase in ratio of skip/full-length *Dmd* transcripts compared to DMSO treatment alone), and near zero levels of endogenous exon 23 exon skip transcripts present in mdx cells [43], [44]. Together this data hints at a possible connection between cell cycle inhibition (most commonly through mitotic arrest) and exon skipping in exogenous and endogenous *DMD* transcripts.

### Exon Skipping Activity Correlates with Mitotic Index

**Figure 3:**
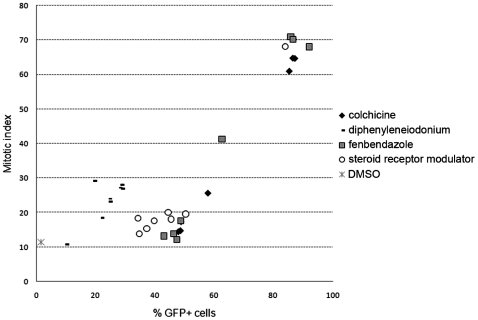
Small molecule enhancers of exon skipping also increase the mitotic index of hE50-GFP C2C12 cells. A positive relationship between mitotic arrest and exon skipping was observed for a subset of small molecule hits in hE50-GFP C2C12 cells transfected with 0.5 µM hE50 AON, and 6 h later treated with 0–10 µM compound. Cells were fixed and analyzed by FACS 24 h after compound addition for exon skipping (%GFP+ cells) and mitotic index (%G2/M cells).

To further test the observation that the ability of a small molecule to induce exon skipping is related to its ability to arrest cells in mitosis, we utilized C2C12 cells stably expressing a human *DMD* minigene construct where treatment with an AON specific to exon 50 triggers splicing out (skipping) of exon 50 from within the coding region of EGFP, thereby restoring its reading frame and increasing GFP signal. Hence the C2C12 *hE50-GFP* cells enabled monitoring of both exon skipping (GFP) and mitotic index status (propidium iodide staining) in the same sample following compound treatment. Reconfirmed purified compounds of each class were tested in 8 point, 3-fold dilutions in C2C12 *hE50-GFP* cells transfected with 0.5 µM hE50 AON. Interestingly, a positive relationship was observed between exon skipping (%GFP positive cells) and mitotic index for the tubulin modulators colchicine and fenbendazole, as well as the flavoenzyme inhibitor diphenyleneiodonium and a steroid hormone receptor modulator (Figure 3). Clearly lacking such a trend in activities were the HDAC inhibitors TSA, scriptaid and dinaline (data not shown). This indicates at least two independent mechanisms of action for the small molecule hits, one that is cell cycle-dependent and another that is not.

### Similar Mechanisms of Action Identified from cDNA and Small Molecule Screens

**Figure 4:**
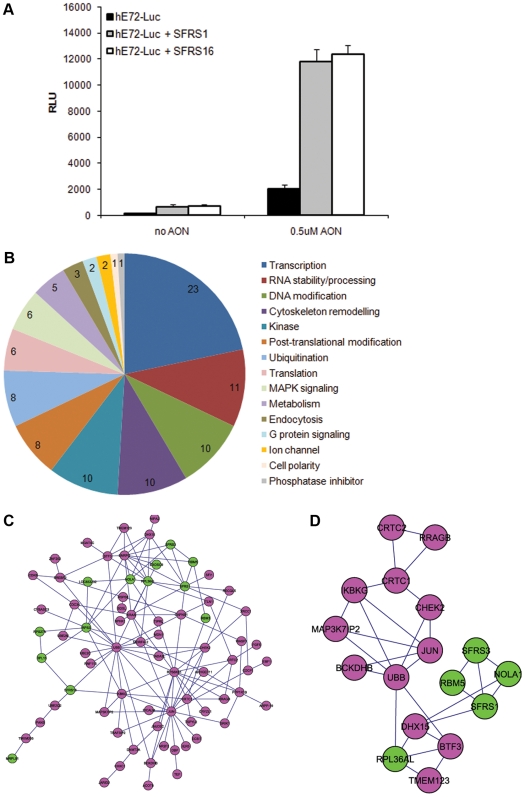
Enhancers of *DMD* exon skipping identified by genome-wide cDNA screening. (A) Luciferase activity (RLU) of hE72-Luc in HEK cells +/−AON and SR protein cDNA positive control constructs 48 h post-transfection. (B) Reconfirmed cDNA hits classified according to known or proposed function. (C) Network cluster diagram of direct protein-protein interactions of reconfirmed cDNA hits (pink) including those with known RNA binding activity (green). RNA binding proteins encoded by hits were EXOSC9, LOC653232, MRPL51, NOLA1, RBM3, RBM5, RPL15, RPL36AL, RPS27A, RPS3, SFRS1, SFRS16, and SFRS3. (D) Sub-network of direct protein-protein interactions at the core of the complex represented in (C), including known RNA binding proteins NOLA1, RBM5, RPL36AL, SFRS1 and SFRS3 (green), and 12 other cDNA hits (pink). See Table S2 for protein details.

In order to better understand the molecular mechanisms involved in exon skipping we performed a genome-wide cDNA overexpression screen. Given the known involvement of serine/arginine-rich (SR) proteins in alternative splicing [45] we first tested a panel of 29 different Origene and MGC cDNA clones encoding SR proteins in co-transfection experiments with hE72-Luc +/−AON. All SR proteins tested induced at least a 2 fold increase in luciferase signal relative to reporter construct alone (data not shown), and were tested in parallel with other clones that did not show such an effect. The best assay window was seen with overexpression of human splicing factor, arginine/serine-rich 1 (SFRS1) (BC033785 in pCMV-SPORT6) or human splicing factor, arginine/serine-rich 16 (SFRS16) (NM_007056.1 in pCMV6-XL5), so these were chosen as positive controls (Figure 4A) in the following screen. Both SFRS1 and SFRS16 gave increased luciferase signals in the presence or absence of hE72 AON, however, a slight synergy of AON and SR protein overexpression was observed (4 fold increase above baseline without AON and 6 fold in the presence of AON).

A collection of ∼17,000 MGC cDNA clones were screened in 384-well format against HEK 293 cells co-transfected with the hE72-Luc(TGA) construct and 0.5 µM hE72 AON. From this screen 143 clones reconfirmed in triplicate (hit rate of 0.83%) by giving ≥2 fold increase in luciferase signal over the plate mean (Table S1). Reconfirmed hits were categorized according to the known function of their protein products (106/143 genes annotated), and as expected for any reporter gene assay, transcription factors were the most common functional group (Figure 4B). Hits with known functional relationships to exon skipping included genes involved in RNA stability or processing (such as Sfrs3 and Rbm3, 4 and 5). Other common gene functions included DNA and chromatin modification, such as histones and the ATP-dependent DNA helicase RecQ5 protein-like 5 (RECQL5). Unexpectedly, but in keeping with the small molecule screening data, clones encoding cell cycle-regulating kinases such as cyclin-dependent kinase 8 (CDK8) and CHK2 checkpoint homolog (Chek2) appeared on the cDNA hit list, together with clones encoding regulators of cytoskeleton remodeling such as tubulin polymerization-promoting protein family member 2 (TPPP2) and Sfi1 homolog, spindle assembly associated (SFI1). In order to highlight the cDNA clones whose overexpression may be directly involved in pre-mRNA processing and exon skipping, we carried out a network analysis using all 135 confirmed human genes (or orthologues of mouse genes) in the database. Considering all interaction pairs among proteins encoded by our confirmed genes, including those proteins known to be associated with RNA based on literature, we obtained a network consisting of 81 proteins and 137 interactions (p<0.001) (Figure 4C), from which we also identified a densely-connected sub-network (Figure 4D). The larger network was found to contain a 23-fold enrichment in components of the 17S U2 snRNP complex (key for splice site selection [46]), 20-fold enrichment in ubiquitin-conjugating enzymes, and 5 to 6-fold enrichment in regulators of cell size and growth (p<0.001), based upon the Gene Ontology (GO) annotation database. The sub-network was more specifically enriched for proteins involved in mRNA splicing and processing and nucleic acid metabolism (proteins in each network are detailed in Table S2). The identification of specific mRNA splicing networks further validated the minigene reporter approach used in this study, and the presence of ubiquitin-conjugating enzymes is in agreement with reports that the ubiquitin pathway is a means of degrading specific splicing factors to promote alternative splicing events [47].

From the list of reconfirmed hits, 45 cDNA clones that were not annotated as encoding general transcription factors (including 9 RNA binding proteins) were selected for further sequence specificity and mechanism of action studies. Genes inducing skip/full-length ratios of >2 after normalization to mock transfected cells in the hE72-Luc qPCR assay (33/45 clones) were regarded as selective enhancers of exon skipping (Table 2). Twenty six of these cDNAs acted in an AON-independent fashion. Genes such as epsin 3 (Epn3), nucleolar protein 8 (NOL8) and RNA binding motif protein 5 (Rbm5) reproducibly induced a higher fold change above baseline in the absence than in the presence of AON. For two of these clones (NOL8 and Rbm5) the same trend was observed in the hE72-Luc luciferase assay. The same 33 clones showing exon skipping enhancement on the hE72-Luc construct were tested for sequence specificity against the hE40-Luc construct. Very few clones enhanced luciferase signal of hE40-Luc, and those that did were generally strong hits in the hE72-Luc assay. Examples of such clones are those encoding RNA binding proteins RBM4 and Rbm5, which are known to alter splicing of genes such as tau [48] and alpha-tropomyosin [49], or caspase 2 [50] and Fas [51], respectively. RBM5 was identified as a member of the protein-protein interaction sub-network described above, as it is one of the better described RNA binding proteins.

**Table 2:** cDNA clones with reconfirmed activity in hE72-Luc assay^[a]^ and specific enhancing effects on exon skipping of the hE72-Luc construct in HEK cells, together with their effects on splicing of endogenous mouse *Dystrophin* transcripts in mdx cells, as determined by qPCR.

Symbol	Gene Name	Genbank Accession	hE72-Luc+AON^[b]^	hE72-Luc^[c]^	hE40-Luc+AON^[b]^	hE40-Luc^[c]^	hE72+AON qPCR^[b]^	hE72 qPCR^[c]^	mdx+AON qPCR^[d]^	mdx qPCR^[e]^
Adck2	AarF domain containing kinase 2	BC069944	8.13	3.74	0.45	0.56	2.09	0.68	1.02	0.53
Atp13a1	ATPase type 13A1	BC138722	7.33	3.92	0.33	0.31	3.82	3.57	1.62	1.75
CAPNS1	Calpain, small subunit 1	BC064998	16.27	4.31	0.60	0.46	2.56	2.11	1.15	0.52
Cdc42ep1	CDC42 effector protein 1	BC083130	11.30	5.24	0.47	0.39	9.89	1.65	1.14	0.81
Chek2	CHK2 checkpoint homolog	BC056617	11.33	6.90	1.44	0.92	2.67	1.90	1.16	0.79
Dnmt3a	DNA methyltransferase 3A, variant 1	BC007466	6.86	2.55	0.87	2.80	4.64	7.56	2.04	1.17
Cdcp1	CUB domain containing protein 1	BC085253	15.17	4.00	0.38	0.31	3.89	3.54	3.12	1.21
Epn3	Epsin 3	BC016454	5.05	2.70	0.26	1.08	2.81	6.87	1.58	2.11
Fgf5	Fibroblast growth factor 5	BC071227	15.74	15.53	0.58	0.78	3.25	1.66	2.24	0.54
GPATC2	G patch domain containing 2	BC042193	7.06	4.59	0.40	1.70	2.40	3.56	2.32	1.46
Gpr39	G protein-coupled receptor 39	BC085285	5.67	3.27	0.30	0.27	2.61	3.42	1.45	0.34
H3f3a	H3 histone, family 3A	BC002268	8.51	6.04	0.58	2.23	5.48	4.68	2.83	1.40
H3F3A	H3 histone, family 3A	BC081561	18.44	18.81	1.63	1.42	3.00	2.45	1.75	1.42
HIST1H4I	Histone cluster 1, H4i	NM_003495	24.47	16.67	1.27	1.01	3.67	1.76	2.87	1.28
Ing1l	Inhibitor of growth family, member 1-like	BC096433	8.65	5.53	1.10	0.79	6.39	3.63	9.06	0.46
Ing4	Inhibitor of growth family, member 4	BC009127	8.04	2.70	1.72	3.79	2.20	2.42	1.59	1.11
EEPD1	Endonuclease/exonuclease/phosphatase family domain containing 1	BC065518	9.58	4.37	0.32	0.34	3.12	2.18	1.29	0.62
Mdn1	Midasin homolog	BC071242	17.70	6.61	1.24	0.88	4.70	3.53	2.60	1.00
MORF4L2	Mortality factor 4 like 2	BC056899	12.40	25.72	7.08	4.09	20.09	6.06	1.80	1.13
Morf4l2	Mortality factor 4 like 2	BC088731	20.50	28.68	10.22	9.22	19.96	8.84	2.12	1.32
Nipa2	Non imprinted in Prader-Willi/Angelman syndrome 2 homolog	BC038499	19.26	8.26	0.75	0.61	2.04	2.58	1.27	0.70
NOL8	Nucleolar protein 8	BC013788	5.05	10.89	0.60	0.45	2.37	4.51	3.11	1.03
RAD1	RAD1 homolog	NM_002853	14.38	11.51	0.77	0.51	2.73	4.41	1.71	0.63
RBM4	RNA binding motif protein 4	BC032735	11.34	12.46	6.38	21.99	4.92	8.32	2.17	1.06
Rbm5	RNA binding motif protein 5	BC031899	15.23	25.12	5.48	3.29	3.53	13.88	2.86	0.89
Rpl15	Ribosomal protein L15	BC091735	10.36	10.78	0.51	0.42	4.66	5.11	2.35	1.19
RPS6KB1	Ribosomal protein S6 kinase, polypeptide 1	BC053365	7.11	21.02	1.03	0.62	3.75	2.70	1.61	1.17
Sfrs3	Splicing factor, arginine/serine-rich 3	BC071196	18.33	3.83	0.58	0.49	2.81	1.58	2.62	0.94
SPHK1	Sphingosine kinase 1	BC030553	9.84	7.61	0.45	1.61	3.29	8.19	2.00	1.00
Stau2	Staufen RNA binding protein homolog 2	AF459099	6.59	4.60	0.82	2.12	3.55	5.65	3.26	1.38
Uaca	Uveal autoantigen with coiled-coil domains and ankyrin repeats	BC033470	5.29	2.54	0.32	0.97	2.87	4.58	3.25	1.27
UBE2C	Ubiquitin-conjugating enzyme E2C, variant 1	BC016292	8.35	2.83	0.88	2.42	4.72	7.17	1.91	1.11
Xrcc1	X-ray repair complementing defective repair in Chinese hamster cells 1	BC085281	28.44	20.26	0.87	0.64	3.06	1.84	1.49	0.53

[a] cDNA clones classed as reconfirmed hits when activity was >2 fold above hE72-Luc alone in an independent experiment to the initial screen; [b] Assay was performed in the presence of 0.5 µM AON and data represents median fold change for Luciferase assays or average fold change for qPCR; [c] Assay was performed in the absence of AON and data represents median fold change for Luciferase assays or average fold change in the normalized skip/full-length ratio for qPCR; [d] mdx-H2K cells were co-transfected with 0.2 µM AON and cDNA clones prior to qPCR analysis and data represents average fold change in the normalized skip/full-length ratio; [e] mdx-HEK cells were transfected with cDNA clones alone and data represents average fold change in the normalized skip/full-length ratio.

Next, the effects of cDNA hits on endogenous mouse *Dmd* splicing were tested in mdx cells (Table 2). Due to the extremely low level of spontaneous *Dmd* exon skipping in mdx cells, only epsin 3 was found to induce a skip/full-length ratio >2 in the absence of AON, while several additional clones showed a weaker activity and skipping ratios >1. Higher levels of splice products could be seen after co-transfection of 0.2 µM AON, and under these conditions overexpression of 8 cDNAs gave a skip/full-length transcript ratio >2. Two of the strongest exon skipping enhancers in the mdx cells of potential therapeutic interest were the nucleolar protein NOL8 and staufen RNA binding protein homolog 2 (Stau2). Both genes code for RNA binding proteins of poorly characterized function, hence they were not identified by data mining of protein-protein interaction databases. NOL8 interacts with at least one DEAD-box RNA helicase DDX47 [52]. Stau2 has a MAPK docking site that is involved in dendritic mRNA transport in neurons [53], [54], and although its function in skeletal muscle is not yet understood, it localizes to the neuromuscular junction, and protein expression levels are increased during myogenic differentiation [55].

### Kinase siRNA Screen Confirms Cell Cycle Regulation of *DMD* Exon Skipping

**Figure 5:**
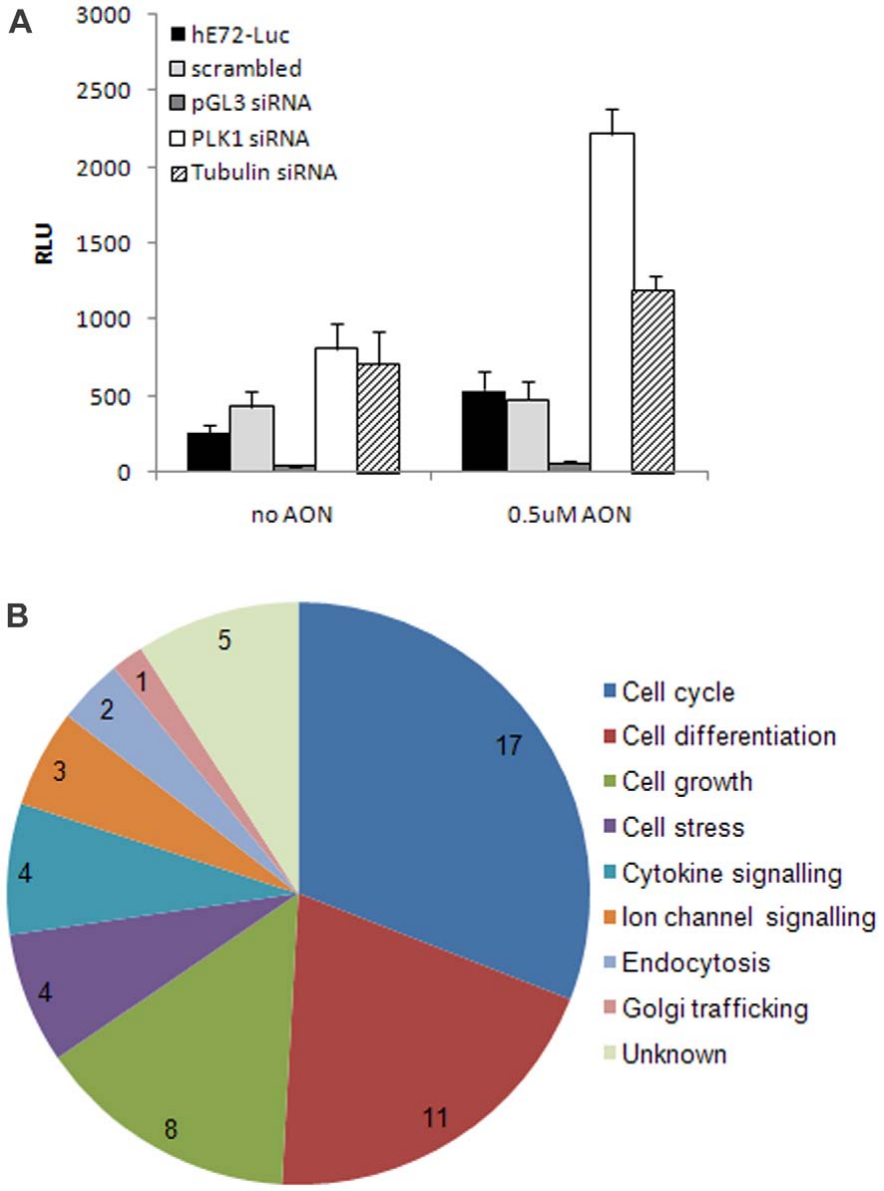
Repressors of *DMD* exon skipping identified by kinome siRNA screening. (A) Luciferase activity (RLU = relative luciferase units) of hE72-Luc in HEK cells +/−AON and *PLK1* and *tubulin* positive control siRNAs 72 h post-transfection. (B) Reconfirmed siRNA hits classified according to known or proposed function.

In order to gain further insight into the mechanism of *DMD* exon skipping, and potentially identify novel targets for drug development, we performed a kinase targeted siRNA screen (4 siRNAs per target for 544 kinases). This screen was performed in 384-well format against HEK 293 cells bulk transfected with the hE72-Luc(TGA) construct in the presence of 0.5 µM hE72 AON, 6 hr prior to siRNA transfection. Since several cell cycle regulators were reconfirmed as hits from our small molecule and cDNA screens, we sought to validate siRNAs targeting tubulin and polo-like kinase 1 (PLK1) as our positive controls. 72 h knockdown of tubulin expression resulted in a 3–4 fold increase in luciferase signal (slightly higher in the presence of AON) and knockdown of PLK1 caused a ∼2.5 fold increase (no additional enhancement in the presence of AON) (Figure 5A). Transfection efficiency was monitored by use of the pGL3 siRNA targeting luciferase and was found to be consistent throughout the screen (data not shown). A hit list of 55 target genes was generated based upon siRNAs that gave ≥2 fold increase in luciferase signal across duplicate plates (Table S3) and these hits were categorized based upon known or predicted functions (Figure 5B). A third of kinase targets with known function are involved in regulation of cell cycle, such as never in mitosis gene a-related kinases (NEKs) and cyclin-dependent kinases (CDKs). Knockdown of NEK10, a gene recently linked to breast cancer susceptibility [56] gave the largest increase in luciferase signal (15 fold above plate mean). As was observed from the small molecule and cDNA screens, this data confirmed the previously observed functional connection between mitotic arrest and enhanced *DMD* exon skipping.

## Discussion

We have used an unbiased screening approach to identify small molecule and genetic regulators of AON-induced exon skipping of *Dystrophin*. Specific enhancement of the ratio of exon skip/full-length transcripts was determined by qPCR analysis, eliminating hits that simply influence transcriptional activation or luciferase stability, a concern for any HTS using luciferase reporters [57]. Ideally a screen for modulators of *DMD* exon skipping would be performed in myotubes, since the tissue most affected in DMD patients is skeletal muscle. However, given the variable nature of myoblast differentiation, this was not technically feasible for a robust HTS assay. The fact that an equivalent number of compounds identified via HTS in HEK 293 cells showed specific enhancement of AON-induced exon skipping of endogenous *Dmd* in the mdx myoblast cellular context as were observed using the hE72-Luc reporter in HEK 293 cells indicates that the mechanisms of action involve ubiquitous molecules. In agreement with our results, Stoilov *et al.* also found that compounds identified via HTS with a minigene reporter construct in HEK 293 cells were capable of specifically enhancing inclusion of exon 10 in endogenous transcripts of *MAPT* [36].

The most potent small molecules identified by our *DMD* exon skipping screen were podophyllotoxin tubulin modulators, and HDAC inhibitors TSA and scriptaid. HDAC2 [58], 4 and 5 [59] have been identified as causative of a higher global deacetylase activity in mdx mice. In addition, both TSA [8] and valproic acid [9] HDAC inhibitors improve morphology and function of skeletal muscle in DMD mouse models - positive effects accredited to the upregulation of follistatin or Akt signaling pathways, respectively. Our data suggests an additional role for HDAC inhibitors in the amelioration of dystrophic phenotype in these studies, that of exon skipping and restoration of *Dystrophin* reading frame.

HDAC inhibitors can act therapeutically in other genetic diseases. TSA increased *SMN2* gene expression in a mouse model of spinal muscular atrophy (SMA), and significantly enhanced exon 7 inclusion (the splicing defect) in *SMN2* transcripts [60]. In addition, M344 specifically enhanced *SMN2* exon 7 inclusion in primary fibroblasts from SMA patients [37], and was proposed to act via upregulation of protein expression of the SR-like splicing factor human transformer 2, beta 1 (Htra2-β1). Sodium butyrate has also been reported to enhance splicing activity in *CFTR*, to correct reading frame of a point mutation associated with cystic fibrosis, and restore chloride channel function [38]. Hence HDAC inhibitors may promote exon inclusion or exclusion (skipping) depending upon the location and sequence of splicing factor binding sites present in the flanking regions. This breadth of action may hinder the usefulness of HDAC inhibitors as therapeutics for genetic disease. However, if DMD protein expression need only be 29% of normal levels to alleviate symptoms of muscle weakness [18], a dosing regimen for HDAC inhibitors may be possible that induces specific exon skipping without substantial off target effects and toxicity seen at high doses in this and other studies [37].

An unexpected finding of this study was the apparent connection between cell cycle progression (particularly mitotic arrest) and exon skipping. Tubulin modulators, cytoskeletal remodeling proteins, and NEK and CDK kinase knockdown were found to stimulate exon skipping in our reporter construct as well as endogenous *Dmd* pre-mRNA. Traditionally it is believed that dissolution of the nuclear envelope and condensation of chromosomes during mitosis inhibits transcription, at least in part, by displacement of the general transcription factor TFIID [61]. However, a recent finding that minor splicing of U12-class introns occurs in the cytoplasm [62], [63] raises the question of how much splicing can occur in mitotic cells? Transcriptional profiling of the cell cycle in human fibroblasts using microarrays identified 227 genes whose expression was enriched during G2/M phase [64]. This data agrees with our finding that *DMD* exon skipping was enhanced by induction of mitotic arrest. Cho *et al.* also found that, in addition to transcripts regulating cell cycle and chromosome segregation, transcripts involved in actin-based cytoskeletal reorganization were up-regulated during G2 phase of the cell cycle [64]. This may explain our observed trend between overexpression of cDNA clones involved in cytoskeleton remodeling and enhanced exon skipping.

DNA intercalators doxifluridine and hoechst were also reconfirmed small molecule hits that enhanced the proportion of *DMD* exon skip transcripts in mdx cells in this study. Another DNA intercalator, aclarubicin, was found to enhance *SMN2* inclusion of exon 7 (resulting in increased SMN2 protein levels) in type I SMA fibroblasts [65]. The mechanism of aclarubicin was proposed to be alteration of SR protein localization. Similarly, phosphatase inhibitor sodium vanadate is proposed to enhance exon 7 inclusion in *SMN2* transcripts via regulation of SR protein phosphorylation, which determines protein sub-localization within the nucleus [66]. Therefore, our surprising finding that induction of mitotic arrest enhances a cell's ability to skip exons in *DMD* may simply be a consequence of the cell cycle-dependent means of regulating RNA binding proteins, and in fact concur with our expected finding that overexpression of RNA binding proteins RBM4, Rbm5 or Sfrs3 promotes *DMD* exon skipping. SR protein kinase 1 (SRPK1) contributes to the majority of SR protein phosphorylation, is 3–5 fold more active in metaphase versus interphase [67], and translocates to the nucleus at the G2/M boundary [68]. In addition to SRPK protein kinases that regulate SR protein phosphorylation and redistribution within the nucleus [69], topoisomerase I can also phosphorylate SR proteins and regulate splicing [70]. Whether DNA intercalators also bind to SR proteins to modify their localization, or act by steric hindrance of SR protein binding to pre-mRNA remains to be determined.

SR proteins play an important role in the regulation of alternative splicing and exon skipping of the *DMD* gene, as highlighted by a case of BMD where exon skipping around a *DMD* exon 27 nonsense mutation results from loss of a purine-rich exonic enhancer site [35]. However the variable phenotypes observed in a family carrying the same nonsense mutation in *DMD* exon 29 (asymptomatic to severe BMD with cardiomyopathy), and partial protein product produced by skipping exon 29, suggest that individuals differ in their ability to activate endogenous exon skipping mechanisms [71]. Such processes do not just apply to DMD, and are not only induced by nonsense mutations. Missense mutations in *SMN2* are enough to abrogate splicing factor arginine/serine-rich 1 (SFRS1/SF2/ASF) binding and promote exon skipping of exon 7 [72] in cases of SMA. Similarly, in cases of frontotemporal dementia with parkinsonism, chromosome 17 type (FTDP-17), several missense, silent and intronic mutations within splicing enhancer and silencer elements in the *MAPT* gene encoding tau can increase or decrease the extent of exon 10 skipping and hence the severity of disease due to tau protein aggregates [73].

Cells maintain a tissue-specific balance of activities of SR proteins and antagonizing hnRNP proteins that can vary during development and mitogenic stress, and these proteins require a specific level of phosphorylation for their activity [74], [75]. SR protein phosphorylation and function can also be regulated by altering chaperones of SRPK1 [76], suggesting that identification of additional binding partners of SRPK proteins, or novel tissue-specific kinases that regulate SR protein activity may prove useful for future therapeutic strategies targeting exon skipping. It would be interesting to further investigate *DMD* pre-mRNA binding sites of some of our novel cDNA screen hits, such as NOL8 and Stau2, as well as the better characterized RBM4 and 5 and Sfrs3, to help understand the process of exon skipping. However, given the importance of SR and RBM proteins for constitutive and alternative splicing during *Drosophila* and mouse development [77]–[79], and maintenance of mammalian cell viability [80], it is unlikely that proteins of these classes hold significant therapeutic potential for DMD unless delivered directly into muscle tissue. However, cancers caused by mutations in SR protein binding sites may benefit from a molecule's ability to both induce mitotic arrest and alter alternative splicing, and there is growing evidence to suggest that altering the balance of SF2/ASF expression regulates malignant transformation via alternative splicing of *Ron* tyrosine kinase receptor and Rac1 GTPase [81].

Here we have used a comprehensive HTS approach utilizing a *DMD* minigene reporter construct to identify novel small molecule and genetic modulators of exon skipping. We confirm previous reports of the importance of balancing the expression levels of splicing factors, by identification of RBM and SR proteins from a genome-wide cDNA screen for regulators of *DMD* exon skipping. In addition, we describe the unexpected relationship between exon skipping and the induction of cell cycle arrest, and propose that this is mediated by regulation of splicing factor distribution and function with G2/M entry. This association with cell cycle was found at the level of small molecule tubulin modulators, siRNAs targeting NEK and CDK kinases, and cDNA clones regulating cytoskeleton remodeling. The fact that one of the first descriptions of HDAC inhibitors' ability to regulate gene expression via chromatin remodeling was in regards to *p21* gene expression, and the subsequent arrest of cells in G1 and G2 phases of the cell cycle [82] also agrees with our finding that small molecules that enhance exon skipping of endogenous and exogenous *Dystrophin* arrest cells in G2 and M phases. It remains to be determined whether HDAC inhibitors are mediating this effect via regulation of SR proteins, or by broader means, but our data does suggest a novel mechanism by which HDAC inhibitors can alleviate symptoms in mdx mice that has yet to be described. The work presented here is a starting point for understanding the endogenous mechanisms of exon skipping within mammalian cells that we hope may contribute to future therapeutics for DMD and other genetic diseases.

## Materials and Methods

### Construct Cloning

The genomic segment spanning exon 71 to exon 73 of human *DMD* was amplified by PCR using NEB *Phusion* polymerase and 200 ng male genomic DNA as template (Promega), generating a 5.6 kb product with *Nco*I restriction sites at both ends (forward: ^5′^TTGCACCATGGTTACTCTGATCAACTTCTG^3′^ and reverse: ^5′^GGATACCATGGTGCTCTCATTAGGAGAGATG^3′^ ). This fragment was cloned into the *Nco*I site immediately upstream of luciferase in the pGL3 promoter vector (Promega), and the ATG start codon of luciferase was mutated to TTG via single primer mutagenesis [83]. Correct orientation and reading frame were confirmed by sequencing. A stop codon mutation was introduced at amino acid position 3427 of exon 72 (numbered according to Leiden muscular dystrophy pages *DMD* reference sequence) [32] using single primer mutagenesis. This resulted in the hE72-Luc(WT), hE72-Luc(TGA), hE72-Luc(TAG), and hE72-Luc(TAA) constructs.

Additional reporter constructs were generated in the same way. For hE40-Luc(WT), hE40-Luc(TGA), hE40-Luc(TAG), and hE40-Luc(TAA), the 4 kb genomic segment spanning exon 39 to exon 41 of human *DMD* was amplified (forward: ^5′^TTGATCCCATGGAAGACAATGAGGGTACTG^3′^ and reverse: ^5′^GTAACCCATGGCAATTTGTGCAAAGTTGAG^3′^ ) and a stop codon introduced at amino acid position 1891. In the case of mE23-Luc(WT) and mE23-Luc(TAA), the 4 kb genomic segment spanning exon 22 to exon 24 of mouse *Dmd* (forward: ^5′^CTTTCCCATGGTTTTTGACACTTTACCACC^3′^ and reverse: ^5′^TACAACCATGGCTCTGCATTGTTTGAGCTG^3′^ ) was amplified, and a TAA stop codon introduced into *Dmd* exon 23 at amino acid position 995, mimicking that of the mdx mouse model [10].

### Antisense Oligonucleotides

AONs with full phosphorothioate backbones and 2′O-Methyl RNA bases specific to the splice sites of human *DMD* exon 72, 40 and 50, and mouse *Dmd* exon 23 were purchased from Integrated DNA Technologies. The following sequences were used: hE72+20+39 ^5′^UGAGUAUCAUCGUGUGAAAG^3′^ ; hE40+127+45 ^5′^UCCUUUCAUCUCUGGGCUC^3′^ ; hE50-19+8 ^5′^AACUUCCUCUUUAACAGAAAAGCAUAC^3′^ ; mE23+2-18 ^5′^GGCCAAACCUCGGCUUACCU^3′^ .

### Tissue Culture

HEK 293 cells were purchased from the American Type Culture Collection (ATCC) and maintained at 37°C with 5% CO_2_ in DMEM (Gibco®) supplemented with 10% fetal bovine serum (Hyclone) and 1X antibiotic-antimycotic (Gibco®). C2C12 cells stably expressing a *DMD hE50-GFP* reporter construct (kindly provided by Dr. Qi Long Lu of the Carolinas HealthCare System) were maintained at 37°C with 5% CO_2_ in DMEM (Gibco®) supplemented with 20% fetal bovine serum (Hyclone) and 1X antibiotic-antimycotic (Gibco®). Conditionally immortal mdx cells generated by intercrossing mdx and H-2K^b^-tsA58 transgenic mice [84] (kind gift from Dr. Qi Long Lu of the Carolinas Healthcare System) were maintained in an undifferentiated myoblast state at 33°C with 5% CO_2_ in DMEM (Gibco®) supplemented with 20% heat-inactivated fetal bovine serum (Hyclone), 2% L-glutamine (Gibco®), 20 U/ml γIFN (Chemicon), 1% chick embryo extract (US Biological) and 1X antibiotic-antimycotic (Gibco®).

### RT-PCR Analysis of Exon Skipping

Spontaneous exon skipping was tested by transient transfection of 1×10^6^ HEK 293 cells in a 9.6 cm^2^ well of a 6-well plate (Greiner) with 1 µg of hE72-Luc or hE40-Luc plasmid DNA and 3 µl of FuGene6 (Roche) as per manufacturer's instructions. Total RNA was extracted 48 h post-transfection using Qiagen RNeasy mini kit columns and quantified using a NanoDrop 1000 spectrophotometer (Thermo Scientific). 200 ng RNA was used as template for cDNA synthesis with random primers and SuperScript® II reverse transcriptase (Invitrogen), as per manufacturer's instructions. 1 µl of the resulting cDNA was used as template for each RT-PCR reaction of 35 cycles with Platinum® *Taq* DNA polymerase (Invitrogen). The primers used were the same as the forward primers used to clone hE72-Luc and hE40-Luc constructs, together with a reverse primer in *luciferase* (pGL2r: ^5′^CTTTATGTTTTTGGCGTCTTCCA^3′^ ). PCR products were visualized by the addition of SYBR® Gold (Invitrogen) to samples prior to electrophoresis at 100 V through 2% agarose (Sigma) dissolved in Tris-acetate-EDTA buffer (Sigma) and density of bands quantified using Alpha Innotech's AlphaEase© FC software, version 3.2.1.

### HTS Transient Luciferase Reporter Gene Assays

HEK 293 cells were transiently transfected in bulk with the *DMD-Luciferase* reporter gene construct, in the presence or absence of a limiting concentration of AON (0.5 µM for hE40 and hE72, and 0.2 µM for mE23). Transfection was achieved with a 3∶1 ratio of FuGene6 (Roche) in Opti-MEM media (Invitrogen), as per manufacturer's instructions, using 1 ng/well for hE72-Luc or 5 ng/well for hE40-Luc and mE23-Luc in 1536-well format, and five times these amounts for 384-well format. Transfection mix was diluted 1∶5 with HEK cells at a density 320,000 cells/ml in growth media lacking antibiotics.

For compound screens, 6 µl transfected cells (1920 cells) were plated per well into 1536-well tissue culture-treated Greiner custom white plates using GNF Systems on-line screening equipment, incubated at 37°C with 5% CO_2_ for 24 h prior to 50 nl compound addition with a PinTool (GNF Systems) to a final compound concentration of 8.3 µM (0.83% DMSO). Luciferase activity was then measured 24 h post-compound addition (48 h post-transfection) by the addition of 3 µl/well Bright-Glo (Promega) and a 60 second luminescence read with a Viewlux™ CCD Imager (Perkin Elmer).

MGC cDNA clones were screened by pre-spotting 40 ng/well cDNA into Greiner white solid bottom 384-well tissue culture plates, then addition of transfection mix containing the *DMD-Luciferase* reporter gene construct +/−AON. HEK 293 cells were then added (8000 cells/well) and cells maintained at 37°C with 5% CO_2_ prior to luciferase activity measurement 48 h post-transfection.

The IDT kinome siRNA library was screened by bulk transfection of the *DMD-Luciferase* reporter gene construct +/− AON into HEK 293 cells 6 h earlier (transfected cells were plated in 175 cm^2^ flasks (Greiner) at a density of 1.5×10^6^ cells/ml). Greiner white solid bottom 384-well tissue culture plates pre-spotted with 14 ng siRNA/well had Lipofectamine RNAiMax (Invitrogen) added in Opti-MEM media (Invitrogen), as per manufacturer's instructions. This transfection mix was diluted with *DMD-Luciferase* transfected cells in growth media lacking antibiotics, to give 8000 cells/well and these cells were maintained at 37°C with 5% CO_2_. Luciferase activity was measured 72 h post-transfection by the addition of Bright-Glo (Promega) and a 60 second luminescence read with a Viewlux™ CCD Imager (Perkin Elmer).

### TaqMan qPCR Analysis

Relative amounts of exon skip and full-length transcripts were quantified using total RNA extracted using Qiagen RNeasy 96 kits in 96-well plate format, SuperScript™III Platinum® One-Step qRT-PCR kits and an Applied Biosystems 7900HT fast real-time PCR system. All TaqMan primers and FAM probes were obtained from Integrated DNA Technologies, and VIC probes were obtained from Applied Biosystems. In all cases standard curves were generated for each 384-well TaqMan plate (Applied Biosystems) using total RNA extracted from HEK 293 cells transiently transfected with hE72-Luc or mE23-Luc constructs. The same samples were used throughout each experiment, allowing comparison of data between different plates.

*DMD* transcript levels were monitored following transient transfection of HEK 293 cells with the hE72-Luc(TGA) minigene reporter construct +/− 0.5 µM AON (40 ng/well reporter and 240,000 cells/well in 24-well Greiner plates), and co-transfection of cDNA clones for 48 h (240 ng/well) or compound treatment for 24 h (0.5% DMSO added 6 h post-transfection), using primer-probe sets specific to the splice junctions of *DMD* exon 71–73 (exon skip) (forward: ^5′^ GTTACTCTGATCAACTTCTG^3′^ , FAM probe: ^5′^ TTTTCCATTTCTGCTAGCGCAGAATCTACTG^3′^ , reverse: ^5′^ CTATCATTTAGATAAGATCCATTG^3′^ ) and exon 72–73 (full-length) (forward: ^5′^ CCTCGTCCCCTCAGCTTTC^3′^ , FAM probe: ^5′^ CACGATGATACTCATTCACGCATTGAACATTATG ^3′^ , reverse ^5′^ CATTGCTGTTTTCCATTTCTGCTA^3′^ ). An initial denaturation step of 10 minutes at 95°C was followed by cDNA synthesis at 52°C for 25 minutes, then products amplified by 40 cycles of 15 seconds at 95°C and 1 minute at 60°C. Data from each primer-probe set was normalized to expression levels of the h36B4 ribosomal protein housekeeping gene for each sample (forward: ^5′^CCACGCTGCTGAACATGC^3′^ , VIC probe: ^5′^AACATCTCCCCCTTCTCCTTTGGGCT^3′^ , reverse: ^5′^TCGAACACCTGCTGGATGAC^3′^ ) and results then expressed as the ratio of normalized skip/full-length transcript levels.

Endogenous mouse *Dmd* exon skip and full-length transcript levels were quantified following 48 h transfection of cDNA clones or 24 h compound treatment (as described above) using mdx myoblast cells +/− 0.2 µM AON and primer-probe sets spanning *Dmd* splice junctions of exon 22–24 (exon skip) (forward: ^5′^TCGGGAAATTACAGAATCACATAAAA^3′^ , FAM probe: ^5′^CCTTACAGAAATGGATGGCTGAAGTTGATGTTT^3′^ , reverse: ^5′^GCAGGCCATTCCTCTTTCAG^3′^ ) and exon 22–23 (full-length) (forward: ^5′^GTTACTGAATATGAAATAATGGAGGAGAGA^3′^ , FAM probe: ^5′^TCGGGAAATTACAGGCTCTGCAAA^3′^ , reverse: ^5′^CCATTTTGTTGCTCTTTCAAAGAA^3′^ ). A denaturation step of 10 minutes at 95°C was followed by cDNA synthesis at 45°C for 40 minutes, then products amplified by 50 cycles of 15 seconds at 95°C and 1 minute at 60°C. Data was normalized as described above.

### High Content Imaging Mitotic Index Assay

HEK 293 cells were plated at a density of 8000 cells/well in custom Greiner clear bottom black 384-well plates. Cells were incubated at 37°C with 5% CO_2_ for 24 h prior to compound addition in 12 point, 3-fold dilutions (highest final concentration 100 µM, 1% DMSO). 24 h post-compound addition cells were fixed by submersion in ice-cold 100% methanol for 5 minutes and stained using a method adapted from Rines *et al.* [85]. Fixative was removed by washing in phosphate-buffered saline (PBS) (Sigma) three times 5 minutes and cells were then incubated in 1.5% BSA/PBS blocking solution for 2 h at room temperature. Blocking solution was removed by washing in PBS three times 5 minutes and antibodies specific for tubulin (1∶1000 FITC conjugated - Sigma) and phosphorylated histone H3 (1∶100 Alexa647 conjugated - BD Biosciences) were added in PBS, together with 0.7 µg/ml hoechst dye 33342 (Invitrogen), and left to incubate overnight at 4°C. Antibodies were removed by washing in PBS three times 5 minutes and cells were imaged with a 10X/0.40 Olympus UPlanSApo objective using an Opera™ high content screening system (Perkin Elmer). The percentage of mitotic cells (mitotic index) was determined by identification of overlapping hoechst, tubulin and phosphorylated histone H3 staining and normalized to that of DMSO treated cells. Known tubulin modulators nocodazole and taxol were included on each plate as positive controls.

### Alamar Blue Toxicity Assay

HEK 293 or mdx cells were plated in Greiner white solid bottom 384-well tissue culture plates in growth media (2000 cells/well in 50 µl) and maintained at 37°C with 5% CO_2_ prior to treatment with 500 nl compound (1% DMSO) or transfected with cDNA clones (as described above) for 48 h prior to addition of equal volume of 1∶5 dilution of alamarBlue® redox indicator (BioSource™) in growth media, to give a final dilution of 1∶10. Cells were maintained for 16–24 h at 37°C with 5% CO_2_ prior to reading fluorescent signal on an Acquest plate reader (LJL Biosystems) using 1000 µs integration time with 530-25 nm excitation and 580-10 nm emission filters. Raw data (counts/second) was normalized to DMSO treated cells and a known toxic compound (staurosporine) was included as a positive control with each experiment.

### FACS Analysis of Cell Cycle and Exon Skipping

C2C12 cells stably expressing an *EGFP* reporter construct interrupted by *DMD* exon 50 (flanked by several hundred base pairs of human *DMD* intronic sequence fused to chicken alpha actin intronic sequence) [86] were plated at a density of 1×10^6^ cells/well in Greiner 6-well plates in the presence of 0.5 µM hE50 AON (transfected using a 3∶1 FuGene 6 to AON ratio, as described above). Compounds were added 6 h post-transfection in 8 point, 3-fold dilutions (highest final concentration 10 µM, 0.5% DMSO) and cells were harvested for FACS analysis 24 h later. In order to preserve GFP signal, cells were fixed with 1X Mirsky's Fixative (National Diagnostics USA) rather than ethanol, and DNA stained with 10 µg/ml propidium iodide (Invitrogen). Data was collected on a BD LSRII and analysis for percentage of cells expressing GFP, and percentage of cells in G2/M phase of the cell cycle was performed using FlowJo software. Nocodazole was included as a positive control for induction of mitotic arrest.

### Protein-Protein Interaction Analysis of cDNA Hits

The list of reconfirmed cDNA hits had all mouse genes converted to their human orthologues, then gene ontology annotation was used to classify proteins with known functions. Multiple human protein-protein interaction databases, such as yeast-2-hybrid databases (Hynet, http://www.prolexys.com) and other literature-based protein-protein interaction databases (STRING, CORUM, Bind, HPRD, MINT, Reactome), were incorporated in the network analysis [87]–[89]. All interaction pairs were collected to form a protein network. Protein networks were evaluated by 1000 permutation simulations and p-values were assigned. The core components in the network were identified by MCODE analysis [90] using Cytoscape V6.2 (http://www.cytoscape.org). Gene Ontology (http://geneontology.org) enrichment analysis was characterized by hypergeometric p-value, as described previously [91].

## Supporting Information

Table S1 cDNA clones with reconfirmed activity in hE72-Luc assay in HEK cells^[a]^, listed according to functional class.
(0.19 MB DOC)

Table S2 Protein-protein interactions of confirmed hits^[a]^ from hE72-Luc cDNA screen.
(0.09 MB DOC)

Table S3 Targets of IDT kinase siRNAs with reconfirmed activity in hE72-Luc assay^[a]^, their accession numbers, functional classes and activity in hE72-Luc assay in HEK cells.
(0.10 MB DOC)
