# Supplementary material for: Identification of Small Molecule and Genetic Modulators of AON-Induced Dystrophin Exon Skipping by High-Throughput Screening
Source: PLoS One. 2009 Dec 17;4(12):e8348. doi: 10.1371/journal.pone.0008348 (PMC2791862; doi:10.1371/journal.pone.0008348)
Supplement: Table S1 — cDNA clones with reconfirmed activity in hE72-Luc assay in HEK cells[a], listed according to functional class. (0.19 MB DOC) [file pone.0008348.s001.doc]

**Table S1.** cDNA clones with reconfirmed activity in hE72-Luc assay in HEK cells^[a]^, listed according to functional class.

| **Symbol** | **Gene Name** | **Genbank Accession** | **Function** |
| --- | --- | --- | --- |
| LIN7C | Lin 7 homolog c | BC004685 | Cell polarity |
| CAPNS1 | Calpain, small subunit 1 | BC064998 | Cytoskeleton remodelling |
| Cdc42ep1 | CDC42 effector protein (Rho GTPase binding) 1 | BC083130 | Cytoskeleton remodelling |
| Elmo3 | Engulfment and cell motility 3 | BC058752 | Cytoskeleton remodelling |
| IER5 | Immediate early response 5 | BC000128 | Cytoskeleton remodelling |
| RAD1 | Human RAD1 homolog | BC035771 | Cytoskeleton remodelling |
| RP1L1 | Retinitis pigmentosa 1 homolog-like 1 | BC031365 | Cytoskeleton remodelling |
| RRAGB | Ras-related GTP binding B, transcript variant RAGB | BC034726 | Cytoskeleton remodelling |
| SFI1 | Sfi1 homolog, spindle assembly associated | BC046221 | Cytoskeleton remodelling |
| TPPP2 | Tubulin polymerization-promoting protein family member 2 | BC038970 | Cytoskeleton remodelling |
| Uaca | Uveal autoantigen with coiled-coil domains and ankyrin repeats | BC033470 | Cytoskeleton remodelling |
| Dnmt3a | DNA methyltransferase 3A, transcript variant 1 | BC007466 | DNA modification |
| EEPD1 | Endonuclease/exonuclease/phosphatase family domain containing 1 | BC065518 | DNA modification |
| H3F3A | H3 histone, family 3A | BC081561 | DNA modification |
| H3f3a | H3 histone, family 3A | BC002268 | DNA modification |
| HIST1H4I | H4 histone family, member M, histone 1, H4i | BC075806 | DNA modification |
| Ing4 | Inhibitor of growth family, member 4 | BC009127 | DNA modification |
| Jmjd2c | Jumonji domain containing 2C | BC020180 | DNA modification |
| Mdn1 | Midasin | BC071242 | DNA modification |
| RECQL5 | ATP-dependent DNA helicase Q5, RecQ protein-like 5 | BC016911 | DNA modification |
| Xrcc1 | X-ray repair complementing defective repair in Chinese hamster cells 1 | BC085281 | DNA modification |
| CYBASC3 | Lysosomal cytochrome b, ascorbate dependent 3 | BC047710 | Endocytosis |
| Epn3 | Epsin 3 | BC016454 | Endocytosis |
| PICALM | Phosphatidylinositol binding clathrin assembly protein, transcript variant 1 | BC048259 | Endocytosis |
| ARHGEF11 | Rho guanine nucleotide exchange factor (GEF) 11 | BC057394 | G protein signaling |
| Gpr39 | G protein-coupled receptor 39 | BC085285 | G protein signaling |
| Grik1 | glutamate receptor, ionotropic, kainate 1 | BC031822 | Ion channel |
| Kcna6 | Potassium voltage-gated channel, shaker-related, subfamily, member 6 | BC048782 | Ion channel |
| Adck2 | AarF domain containing kinase 2 | BC069944 | Kinase |
| CDK8 | Cyclin-dependent kinase 8 | BC025046 | Kinase |
| Chek2 | CHK2 checkpoint homolog | BC056617 | Kinase |
| Hspb8 | Heat shock 27kDa protein 8 | BC011219 | Kinase |
| Ikbkg | Inhibitor of kappaB kinase gamma | BC021431 | Kinase |
| PAN3 | PAN3 polyA specific ribonuclease subunit | BC061486 | Kinase |
| RPS6KB1 | Ribosomal protein S6 kinase, 70kDa, polypeptide 1 | BC053365 | Kinase |
| SCYL1BP1 | SCY1-like 1 binding protein 1, NTKL-binding protein 1 | BC064945 | Kinase |
| SPHK1 | Sphingosine kinase 1 | BC030553 | Kinase |
| Trib2 | Tribbles homolog 2 | BC037387 | Kinase |
| ECSIT | ECSIT homolog, signaling intermediate in Toll pathway-evolutionarily conserved | BC004583 | MAPK signaling |
| Fgf5 | Fibroblast growth factor 5 | BC071227 | MAPK signaling |
| KRAS2 | v-Ki-ras2 Kirsten rat sarcoma viral oncogene homolog, transcript variant b | BC013572 | MAPK signaling |
| Map3k7ip2 | Mitogen-activated protein kinase kinase kinase 7 interacting protein 2 | BC004813 | MAPK signaling |
| TIPRL | TIP41, TOR signaling pathway regulator-like | BC058250 | MAPK signaling |
| TRAF3IP2 | TRAF3 interacting protein 2 | BC096483 | MAPK signaling |
| ACOT8 | Acyl-CoA thioesterase 8, peroxisomal acyl-CoA thioesterase 1 | BC005792 | Metabolism |
| BCKDHB | Branched chain keto acid dehydrogenase E1, beta polypeptide | BC040139 | Metabolism |
| Cyp2c70 | Cytochrome P450, family 2, subfamily c, polypeptide 70 | BC016494 | Metabolism |
| HTRA3 | High-temperature requirement factor A3 serine peptidase 3 | BC035717 | Metabolism |
| Sdsl | Serine dehydratase-like | BC022601 | Metabolism |
| Ppp1r1b | Protein phosphatase 1, regulatory (inhibitor) subunit 1B | BC011122 | Phosphatase inhibitor |
| Golph3l | Golgi phosphoprotein 3-like | BC047147 | Post-translational modifications |
| Gosr2 | Golgi SNAP receptor complex member 2 | BC051253 | Post-translational modifications |
| MGAT4C | Mannosyl (alpha-1,3-)-glycoprotein beta-1,4-N-acetylglucosaminyltransferase, isozyme C | BC046987 | Post-translational modifications |
| MORF4L2 | Mortality factor 4 like 2 | BC056899 | Post-translational modifications |
| Morf4l2 | Mortality factor 4 like 2 | BC088731 | Post-translational modifications |
| SCFD1 | Sec1 family domain containing 1, transcript variant 1 | BC017734 | Post-translational modifications |
| ZDHHC12 | Zinc finger, DHHC domain containing 12 | BC048251 | Post-translational modifications |
| ZDHHC13 | Zinc finger, DHHC domain containing 13, transcript variant 1 | BC056152 | Post-translational modifications |
| Arpp19 | cAMP-regulated phosphoprotein 19 | BC040206 | RNA stability/processing |
| Cryzl1 | Crystallin, zeta (quinone reductase)-like 1 | BC019387 | RNA stability/processing |
| DHX15 | DEAH (Asp-Glu-Ala-His) box polypeptide 15 | BC003745 | RNA stability/processing |
| Exosc9 | Exosome component 9 | BC052156 | RNA stability/processing |
| NOL8 | Nucleolar protein Nop132, nucleolar protein 8 | BC013788 | RNA stability/processing |
| Nola1 | Nucleolar protein family A, member 1 | BC021873 | RNA stability/processing |
| Rbm3 | RNA binding motif protein 3 | BC086491 | RNA stability/processing |
| RBM4 | RNA binding motif protein 4 | BC032735 | RNA stability/processing |
| Rbm5 | RNA binding motif protein 5 | BC031899 | RNA stability/processing |
| Sfrs3 | Splicing factor, arginine/serine-rich 3 (SRp20) | BC071196 | RNA stability/processing |
| Stau2 | Staufen RNA binding protein homolog 2 | BC025118 | RNA stability/processing |
| AEBP2 | AE binding protein 2 | BC025467 | Transcription |
| Btf3 | Basic Transcription 3 | BC064010 | Transcription |
| Cdca4 | Cell division cycle associated 4 | BC055824 | Transcription |
| CRTC1 | CREB regulated transcription coactivator 1, mucoepidermoid carcinoma translocated 1 | BC028050 | Transcription |
| CRTC2 | CREB regulated transcription coactivator 2 | BC053562 | Transcription |
| CXXC1 | CXXC finger 1 (PHD domain) | BC014940 | Transcription |
| Dbp | D site albumin promoter binding protein | BC018323 | Transcription |
| DIP2A | DIP2 disco-interacting protein 2 homolog A | BC033718 | Transcription |
| EBF | Early B-cell factor | BC038805 | Transcription |
| ING2 | Inhibitor of growth family, member 2 (alias Ing1l, p33Ing2) | BC096433 | Transcription |
| JARID2 | Jumonji, AT rich interactive domain 2 | BC046246 | Transcription |
| Jun | Jun oncogene | BC002081 | Transcription |
| NR2F2 | Nuclear receptor subfamily 2, group F, member 2 | BC042897 | Transcription |
| Rnf4 | Ring finger protein 4 | BC003282 | Transcription |
| Sox11 | SRY-box containing gene 11 | BC078643 | Transcription |
| Srebf2 | Sterol regulatory element binding factor 2 | BC048071 | Transcription |
| Srebf2 | Sterol regulatory element binding factor 2 | BC069989 | Transcription |
| TADA3L | Transcriptional adaptor 3 (NGG1 homolog)-like | BC023879 | Transcription |
| TEF | Thyrotrophic embryonic factor | BC042476 | Transcription |
| TFAP2C | Transcription AP-2 gamma (activating enhancer binding protein 2 gamma) | BC035664 | Transcription |
| TSPYL2 | TSPY-like 2, CDA1 (cell division autoantigen-1) | BC030334 | Transcription |
| Zfp202 | Zinc finger protein 202 | BC049772 | Transcription |
| ZXDC | ZXD family zinc finger C | BC003332 | Transcription |
| Mrpl51 | Mitochondrial ribosomal protein L51 | BC021535 | Translation |
| Rpl15 | Ribosomal protein L15 | BC091735 | Translation |
| RPL15 | Ribosomal protein L15 | BC014837 | Translation |
| RPL32P3 | Ribosomal protein L32 pseudogene 3 | BC053996 | Translation |
| Rps3 | Ribosomal protein S3 | BC010721 | Translation |
| TRSPAP1 | tRNA selenocysteine associated protein 1 | BC055454 | Translation |
| Cdc34 | Cell division cycle 34 homolog | BC094502 | Ubiquitination |
| RNF115 | Rabring 7, ring finger protein 115, zinc finger protein 364 | BC054049 | Ubiquitination |
| RPS27A | Ribosomal protein S27a | BC053371 | Ubiquitination |
| UBB | Ubiquitin B | BC038999 | Ubiquitination |
| UBE2C | Ubiquitin-conjugating enzyme E2C, transcript variant 1 | BC016292 | Ubiquitination |
| Ube2m | Ubiquitin-conjugating enzyme E2M (UBC12 homolog) | BC026581 | Ubiquitination |
| UBE2Q2 | Ubiquitin-conjugating enzyme E2Q family member 2 | BC034342 | Ubiquitination |
| UBR5 | Ubiquitin protein ligase E3 component n-recognin 5 | BC057458 | Ubiquitination |
| 2310040C09Rik | RIKEN cDNA 2310040C09 gene | BC085279 | Unknown |
| 2810047C21Rik | RIKEN cDNA 2810047C21 gene | BC071238 | Unknown |
| ANKRD7 | Ankyrin repeat domain 7 | BC032799 | Unknown |
| Atp13a1 | ATPase type 13A1 | BC049817 | Unknown |
| C10orf30 | Chromosome 10 open reading frame 30 | BC096430 | Unknown |
| C14orf106 | Chromosome 14 open reading frame 106 | BC065544 | Unknown |
| C15orf29 | Chromosome 15 open reading frame 29 | BC003216 | Unknown |
| C15orf29 | Chromosome 15 open reading frame 29 | BC080661 | Unknown |
| C17orf45 | Chromosome 17 open reading frame 45 | BC040159 | Unknown |
| C1orf130 | Chromosome 1 open reading frame 130 | BC039748 | Unknown |
| CCDC117 | Coiled-coil domain containing 117 | BC034558 | Unknown |
| CDCP1 | Alias CD318, CUB domain containing protein 1 | BC085253 | Unknown |
| CRISPLD1 | Cysteine-rich secretory protein LCCL domain containing 1, Cocoacrisp protein | BC040768 | Unknown |
| FLJ11506 | Alpha- and gamma-adaptin-binding protein p34 | BC047026 | Unknown |
| FLYWCH1 | FLYWCH-type zinc finger 1 | BC038031 | Unknown |
| GIYD1 | GIY-YIG domain containing 1 | BC055909 | Unknown |
| GPATC2 | G patch domain containing 2 | BC042193 | Unknown |
| HIG2 | Hypoxia-inducible protein 2 | BC008573 | Unknown |
| HYPK | Huntingtin interacting protein K | BC021588 | Unknown |
| LOC340602 | Similar to CG32656-PA | BC046248 | Unknown |
| ND^b]^ | Clone IMAGE:4151631 | BC013641 | Unknown |
| ND^b]^ | Clone IMAGE:4509171 | BC012487 | Unknown |
| ND^b]^ | Hypothetical protein FLJ12649 | BC028177 | Unknown |
| Nipa2 | Non imprinted in Prader-Willi/Angelman syndrome 2 homolog | BC038499 | Unknown |
| RNF145 | Ring finger protein 145 | BC040799 | Unknown |
| Sh3tc2 | SH3 domain and tetratricopeptide repeats 2 | BC049856 | Unknown |
| Tbrg3 | Transforming growth factor beta regulated gene 3 | BC095996 | Unknown |
| THAP10 | THAP domain containing 10 | BC027857 | Unknown |
| TMEM123 | Transmembrane protein 123 | BC086319 | Unknown |
| Tmem14a | Transmembrane protein 14A | BC060034 | Unknown |
| TMEM205 | Transmembrane protein 205, UNQ501, MBC3205 | BC064948 | Unknown |
| TMEM77 | Transmembrane protein 77 | BC047025 | Unknown |
| TMEM81 | Transmembrane protein 81 | BC095954 | Unknown |
| WDR89 | WD repeat domain 89 | BC010698 | Unknown |
| Zfp101 | Zinc finger protein 101 | BC002058 | Unknown |
| Zfp54 | Zinc finger protein 54 | BC080782 | Unknown |
| ZNF174 | Zinc finger protein 174 | BC000876 | Unknown |

[a] cDNA clones classed as reconfirmed hits when activity was >2 fold above hE72-Luc alone in an independent experiment to the initial screen; [b] ND = not determined.
