# Supplementary material for: Identification of Small Molecule and Genetic Modulators of AON-Induced Dystrophin Exon Skipping by High-Throughput Screening
Source: PLoS One. 2009 Dec 17;4(12):e8348. doi: 10.1371/journal.pone.0008348 (PMC2791862; doi:10.1371/journal.pone.0008348)
Supplement: Table S2 — Protein-protein interactions of confirmed hits[a] from hE72-Luc cDNA screen. (0.09 MB DOC) [file pone.0008348.s002.doc]

**Table S2.** Protein-protein interactions of confirmed hits^[a]^ from hE72-Luc cDNA screen.

| **NETWORK** |  | **SUB-NETWORK** |  |
| --- | --- | --- | --- |
| **PROTEIN SYMBOL** | **NO. OF INTERACTIONS** | **PROTEIN SYMBOL** | **NO. OF INTERACTIONS** |
| ACOT8 | 1 |  |  |
| ARHGEF11 | 3 |  |  |
| ARPP-19 | 1 |  |  |
| BCKDHB | 4 | BCKDHB | 2 |
| BTF3 | 7 | BTF3 | 4 |
| CAPNS1 | 7 |  |  |
| CDC34 | 4 |  |  |
| CDCP1 | 3 |  |  |
| CDK8 | 2 |  |  |
| CHEK2 | 7 | CHEK2 | 3 |
| CRTC1 | 5 | CRTC1 | 4 |
| CRTC2 | 5 | CRTC2 | 2 |
| CRYZL1 | 1 |  |  |
| CXXC1 | 2 |  |  |
| CYBASC3 | 1 |  |  |
| DBP | 2 |  |  |
| DHX15 | 6 | DHX15 | 4 |
| DNMT3A | 3 |  |  |
| EBF1 | 1 |  |  |
| ECSIT | 1 |  |  |
| EXOSC9^[b]^ | 2 |  |  |
| FGF5 | 1 |  |  |
| GRIK1 | 2 |  |  |
| HSPB8 | 3 |  |  |
| IER5 | 1 |  |  |
| IKBKG | 9 | IKBKG | 4 |
| JARID2 | 1 |  |  |
| JMJD2C | 2 |  |  |
| JUN | 16 | JUN | 5 |
| KRAS | 10 |  |  |
| LOC653232^[b]^ | 2 |  |  |
| MAP3K7IP2 | 3 | MAP3K7IP2 | 3 |
| MDN1 | 1 |  |  |
| MGAT4C | 1 |  |  |
| MORF4L2 | 7 |  |  |
| MRPL51^[b]^ | 1 |  |  |
| NIPA2 | 1 |  |  |
| NOLA1^[b]^ | 8 | NOLA1^[b]^ | 3 |
| NR2F2 | 1 |  |  |
| PAN3 | 2 |  |  |
| PICALM | 2 |  |  |
| PPP1R1B | 5 |  |  |
| RBM3^[b]^ | 2 |  |  |
| RBM5^[b]^ | 4 | RBM5^[b]^ | 2 |
| RECQL5 | 2 |  |  |
| RNF115 | 1 |  |  |
| RPL15^[b]^ | 3 |  |  |
| RPL36AL^[b]^ | 10 | RPL36AL^[b]^ | 4 |
| RPS27A^[b]^ | 2 |  |  |
| RPS3^[b]^ | 9 |  |  |
| RPS6KB1 | 14 |  |  |
| RRAGB | 3 | RRAGB | 2 |
| SDSL | 1 |  |  |
| SFI1 | 1 |  |  |
| SFRS1^[b]^ | 10 | SFRS1^[b]^ | 4 |
| SFRS16^[b]^ | 4 |  |  |
| SFRS3^[b]^ | 4 | SFRS3^[b]^ | 3 |
| SNRPG | 11 |  |  |
| SPHK1 | 1 |  |  |
| SREBF2 | 5 |  |  |
| TADA3L | 4 |  |  |
| TEF | 1 |  |  |
| TIPRL | 2 |  |  |
| TLN1 | 3 |  |  |
| TMEM123 | 3 | TMEM123 | 2 |
| TMEM205 | 2 |  |  |
| TRAF3IP2 | 2 |  |  |
| TSPYL2 | 1 |  |  |
| UBB | 18 | UBB | 7 |
| UBE2C | 1 |  |  |
| UBE2M | 2 |  |  |
| UBE2Q2 | 2 |  |  |
| WASF1 | 2 |  |  |
| XRCC1 | 3 |  |  |
| ZNF202 | 1 |  |  |

[a] cDNA clone classed as reconfirmed hit when luciferase activity was >2 fold above hE72-Luc alone in an independent experiment; [b] Known RNA-binding protein.
