# Supplementary material for: Identification of Small Molecule and Genetic Modulators of AON-Induced Dystrophin Exon Skipping by High-Throughput Screening
Source: PLoS One. 2009 Dec 17;4(12):e8348. doi: 10.1371/journal.pone.0008348 (PMC2791862; doi:10.1371/journal.pone.0008348)
Supplement: Table S3 — Targets of IDT kinase siRNAs with reconfirmed activity in hE72-Luc assay[a], their accession numbers, functional classes and activity in hE72-Luc assay in HEK cells. (0.10 MB DOC) [file pone.0008348.s003.doc]

**Table S3.** Targets of IDT kinase siRNAs with reconfirmed activity in hE72-Luc assay^[a]^, their accession numbers, functional classes and activity in hE72-Luc assay in HEK cells.

| **Symbol** | **Gene Name** | **Genbank Accession** | **Function** | **hE72Luc+AON Average Fold Change** |
| --- | --- | --- | --- | --- |
| BUB1 | Budding uninhibited by benzimidazoles 1 | NM_004336 | Cell cycle | 5.14 |
| BUB1B | Budding uninhibited by benzimidazoles 1 homolog beta | NM_001211 | Cell cycle | 2.20 |
| CDC2L6 | Cell division cycle 2-like 6 | NM_015076 | Cell cycle | 2.28 |
| CDK3 | Cyclin-dependent kinase 3 | NM_001258 | Cell cycle | 2.57 |
| CDKL5 | Cyclin-dependent kinase-like 5 | NM_003159 | Cell cycle | 2.14 |
| LYK5 | Protein kinase LYK5 | NM_001003788 | Cell cycle | 2.93 |
| ND^[b]^ | Similar to Serine/threonine-protein kinase PLK1 | XM_498286 | Cell cycle | 2.99 |
| NEK1 | NIMA (never in mitosis gene a)-related kinase 1 | NM_012224 | Cell cycle | 2.17 |
| NEK10 | NIMA (never in mitosis gene a)- related kinase 10 | NM_001031741 | Cell cycle | 15.03 |
| NEK11 | NIMA (never in mitosis gene a)- related kinase 11 | NM_024800 | Cell cycle | 2.73 |
| NEK5 | NIMA (never in mitosis gene a)-related kinase 5 (NEK5) | XM_292160 | Cell cycle | 2.36 |
| NEK7 | NIMA (never in mitosis gene a)-related kinase 7 | NM_133494 | Cell cycle | 2.51 |
| NEK8 | NIMA (never in mitosis gene a)- related kinase 8 | NM_178170 | Cell cycle | 5.25 |
| PFTK1 | PFTAIRE protein kinase 1 | NM_012395 | Cell cycle | 2.67 |
| SCYL1 | SCY1-like 1 | NM_020680 | Cell cycle | 2.50 |
| SMG1 | PI-3-kinase-related kinase | NM_015092 | Cell cycle | 2.21 |
| TLK1 | Tousled-like kinase 1 | NM_012290 | Cell cycle | 4.46 |
| CAMK1 | Calcium/calmodulin-dependent protein kinase 1 | NM_003656 | Cell differentiation | 7.93 |
| CSNK1A1 | Casein kinase 1, alpha 1 | NM_001025105 | Cell differentiation | 3.16 |
| DDR2 | Discoidin domain receptor family member 2 | NM_001014796 | Cell differentiation | 2.97 |
| EPHA4 | EPH receptor A4 | NM_004438 | Cell differentiation | 3.06 |
| LIMK2 | LIM domain kinase 2 | NM_001031801 | Cell differentiation | 2.41 |
| MYO3B | Myosin IIIB, variant 2 | NM_138995 | Cell differentiation | 2.31 |
| NLK | Nemo-like kinase | NM_016231 | Cell differentiation | 2.24 |
| NTRK3 | Neurotrophic tyrosine kinase receptor type 3 | NM_001007156 | Cell differentiation | 2.57 |
| NYDSP25 | Protein kinase NYD-SP25 | NM_001001875 | Cell differentiation | 3.24 |
| TNIK | TRAF2 and NCK interacting kinase | NM_015028 | Cell differentiation | 3.92 |
| TRIB1 | Tribbles homolog 1 | NM_025195 | Cell differentiation | 7.40 |
| ERBB3 | V-erb-b2 erythroblastic leukemia viral oncogene homolog 3 | NM_001005915 | Cell growth | 8.50 |
| FGFR3 | Fibroblast growth factor receptor 3 | NM_000142 | Cell growth | 4.62 |
| MAP2K1 | Mitogen-activated protein kinase kinase 1 | NM_002755 | Cell growth | 2.45 |
| MAP2K4 | Mitogen-activated protein kinase kinase 4 | NM_003010 | Cell growth | 9.90 |
| MKNK2 | MAP kinase interacting serine/threonine kinase 2 | NM_017572 | Cell growth | 8.86 |
| PKN3 | Protein kinase N3 | NM_013355 | Cell growth | 2.47 |
| RPS6KL1 | Ribosomal protein S6 kinase-like 1 | NM_031464 | Cell growth | 2.27 |
| STK24 | Serine/threonine kinase 24, variant 2 | NM_001032296 | Cell growth | 2.61 |
| GUCY2C | Guanylate cyclase 2C | NM_004963 | Cell stress | 2.56 |
| MAP4K5 | Mitogen-activated protein kinase kinase kinase kinase 5 | NM_006575 | Cell stress | 2.94 |
| MINK1 | Misshapen-like kinase 1 | NM_001024937 | Cell stress | 5.82 |
| PINK1 | PTEN induced putative kinase 1 | NM_032409 | Cell stress | 2.83 |
| IRAK1 | Interleukin-1 receptor-associated kinase 1 | NM_001025242 | Cytokine signalling | 5.06 |
| JAK3 | Janus kinase 3 | NM_000215 | Cytokine signalling | 2.31 |
| MAP3K14 | Mitogen-activated protein kinase kinase kinase 14 | NM_003954 | Cytokine signalling | 2.17 |
| RIPK2 | Receptor-interacting serine-threonine kinase 2 | NM_003821 | Cytokine signalling | 2.38 |
| AAK1 | AP2 associated kinase 1 | NM_014911 | Endocytosis | 3.26 |
| SCYL2 | SCY1-like 2 | NM_017988 | Endocytosis | 5.75 |
| PRKD2 | Protein kinase D2 | NM_016457 | Golgi trafficking | 3.29 |
| ALPK1 | Alpha-kinase 1 | NM_025144 | Ion channel signalling | 2.43 |
| PRKCH | Protein kinase C eta | NM_006255 | Ion channel signalling | 2.84 |
| WNK4 | WNK lysine deficient protein kinase 4 | NM_032387 | Ion channel signalling | 2.89 |
| ALPK2 | Alpha-kinase 2 | NM_052947 | Unknown | 3.84 |
| BCR | Breakpoint cluster region | NM_004327 | Unknown | 13.88 |
| MAP3K15 | Mitogen-activated protein kinase kinase kinase 15 | NM_001001671 | Unknown | 2.32 |
| PSKH2 | Protein serine kinase H2 | NM_033126 | Unknown | 4.57 |
| VRK3 | Vaccinia related kinase 3, variant 2 | NM_001025778 | Unknown | 4.34 |

[a] siRNA classed as reconfirmed when luciferase activity was >2 fold above baseline in duplicate plates; [b] Not determined.
